# *Desulfosporosinus* and *Acididesulfobacillus* dominate an acidophilic sulfate-reducing bacteria consortium during acid mine drainage bioremediation

**DOI:** 10.1128/aem.00308-26

**Published:** 2026-04-24

**Authors:** Luis Felipe Valdez-Nuñez, Idelso Jamín Chávez, Fatih Sekerci, Diana Ayala-Muñoz, Daniel Straub, Andreas Kappler, Stefan Fischer, Muammar Mansor

**Affiliations:** 1 Research Institute on Mines and the Environment (RIME), University of Québec in Abitibi-Témiscamingue (UQAT), Rouyn-Noranda, Quebec, Canada; 2 Biotechnology, Department of Biological Sciences, Universidad Nacional de Cajamarca, Cajamarca, Peru; 3 Geomicrobiology, Department of Geosciences, University of Tübingen, Tübingen, Germany; 4 Biotechnology Engineering, Department of Engineering and Applied Sciences, Universidad de Las Américas, Quito, Ecuador; 5 University of Tübingen, Quantitative Biology Center (QBiC), Tübingen, Germany; 6 M3 Research Center, Medical Faculty, University of Tübingen, Tübingen, Germany; 7 Cluster of Excellence (EXC 3121): TERRA – Terrestrial Geo-Biosphere Interactions in a Changing World, University of Tübingen, Tübingen, Germany.; 8 Tübingen Structural Microscopy Core Facility, University of Tübingen, Tübingen, Germany; Georgia Institute of Technology, Atlanta, Georgia, USA

**Keywords:** acid mine drainage, acidophilic, sulfate-reducing bacteria, consortium, mineral formation, *Acididesulfobacillus*, *Desulfosporosinus*

## Abstract

Acid mine drainage (AMD) is an environmental threat due to its low pH and high metal content. Biological treatment of AMD using acidophilic sulfate-reducing bacteria (aSRB) represents a potential solution for this problem, but their substrate specificity and low tolerance to extreme acidity (pH ≤3.0) and toxic metals limit their application. Here, we used an indigenous aSRB-containing consortium to remove metals and neutralize a synthetic AMD (sAMD) system starting at pH 2.9. The consortium was enriched from acidic sediments of an abandoned mine tunnel in Peru. A bioremediation experiment (pH 2.9) was set up with Fe^2+^ (40.25 mM), Al^3+^ (5.39 mM), and Zn^2+^ (3.97 mM) as the main dissolved metals. Glycerol and yeast extract were used as carbon sources. Physicochemical parameters, mineral formation, microbial communities, and dissolved metals were monitored for 160–200 days. At the end of the incubation, the final pH reached 6.1 and 100% of Zn^2+^, >99% of Fe^2+^, and >94% of Al^3+^ were removed by the aSRB consortium as X-ray diffraction-amorphous minerals. The aSRB *Desulfosporosinus* and *Acididesulfobacillus* dominated the bioremediation experiment. Two high-quality metagenome-assembled genomes taxonomically affiliated to the aforementioned aSRB showed metabolic potential related to sulfur compounds reduction as well as to organic carbon degradation (e.g., glycerol and acetate). Differences related to carbon degradation during AMD bioremediation suggest a synergy between *Acididesulfobacillus* and *Desulfosporosinus*, thus avoiding toxic waste product accumulation. Overall, we obtained a novel aSRB-containing microbial consortium that can be used for acidity neutralization and metal removal, suitable for more robust AMD treatment technologies.

**IMPORTANCE:**

Acid mine drainage (AMD) remains one of the biggest environmental challenges of the mining industry. Treatment technologies based on the application of microbial consortia are gaining popularity, taking advantage of synergistic interactions between different species to widen substrate specificity and to limit toxicity. Our research work here shows two acidophilic sulfate-reducing bacteria, *Desulfosporosinus* and *Acididesulfobacillus*, working together in AMD bioremediation. *Desulfosporosinus* initiated sulfate reduction at pH ~3.0 with glycerol as the carbon source and acetate as the waste product. Once pH rose to ~4.0, *Acididesulfobacillus* continued with sulfate reduction with acetate as a carbon source, thus avoiding acetate accumulation and cell toxicity. In the end, this synergistic interaction neutralized acidic pH and removed metals to a great extent, making it suitable for biological treatment of AMD.

## INTRODUCTION

Acid mine drainage (AMD) contains toxic waste derived from mining activities and is characterized by its low pH (≤3.0) and high metal and sulfate content (1). AMD is generated by a combination of abiotic and biotic processes in which sulfide ores (e.g., pyrite FeS_2_) are oxidized by oxygen and Fe^3+^, catalyzed by chemolithoautotrophic bacteria such as *Acidithiobacillus* and *Leptospirillum* at pH ≤3.0 (2–4). AMD is responsible for metal pollution once it enters the ecosystems, generating serious concerns for the environment and human health (1). Current AMD treatment relies on the addition of chemicals (e.g., CaCO_3_) to neutralize the acidity and to precipitate metals, but the increasing costs of these reagents affect their sustainability. AMD is still one of the biggest environmental challenges of the mining industry.

For several years, the use of acidophilic sulfate-reducing bacteria (aSRB) has been explored for AMD bioremediation (5, 6). aSRB perform dissimilatory sulfate reduction, an anaerobic step of the sulfur cycle in which hydrogen sulfide is the end product. Organic matter is also oxidized during this metabolism to simple organic compounds (e.g., acetate) or inorganic carbon (i.e., bicarbonate) (7, 8). Both hydrogen sulfide and bicarbonate react with toxic metals and increase the pH in the AMD systems, promoting metal precipitation and acidity neutralization (5, 8). *Desulfosporosinus* is the main acidophilic sulfate reducer genus reported in several AMD treatment experiments (9–11). Nonetheless, limitations related to toxicity to a pH lower than 3.6 (i.e., *Desulfosporosinus acidiphilus* [12]) render the need to search for novel strategies to prompt a more robust and efficient AMD treatment.

The use of an aSRB consortia instead of pure cultures is a rising alternative for AMD bioremediation. Metabolic redundancy and synergistic interactions between different microbial species in a consortium may allow aSRB survival by creating a fast metabolic flow of organic and inorganic waste products and ideal pH conditions for cell growth and activity. Several microbial consortia have been obtained and tested for AMD treatment (e.g., *Desulfosporosinus acididurans* and *Peptococcaceae* strain CEB3), especially at pH ≥4.0 (pH during reactor performance), reaching high levels of metal precipitation (e.g., >90% of Cu, Zn), sulfate removal >50%, and acidity neutralization (up to pH 6.0) at the end of the treatment (9, 13). However, the use of aSRB microbial consortia to treat highly acidic AMD (pH ≤3.0) is still scarce. Using an aSRB consortium for AMD treatment may not only improve the robustness of the microbial communities throughout time but also promote more efficient AMD bioremediation by enhancing sulfate reduction, metal precipitation, and acidity neutralization (8).

In this study, an aSRB consortium was enriched using acidic sediments from a mining tunnel located in Hualgayoc (Cajamarca), Peru, and tested for synthetic AMD (sAMD, pH 2.9) bioremediation. During the experiment, physicochemical, mineralogical, and microbial community analyses were performed. Our results indicate that the aSRB consortium was able to precipitate metals such as Fe and Zn to >99% and Al to >94% extent while neutralizing the acidity (up to pH 6.1) in the sAMD. This research highlights the application of an indigenous aSRB-containing consortium for AMD bioremediation.

## MATERIALS AND METHODS

### Mining tunnel and sample collection

**Fig 1:**
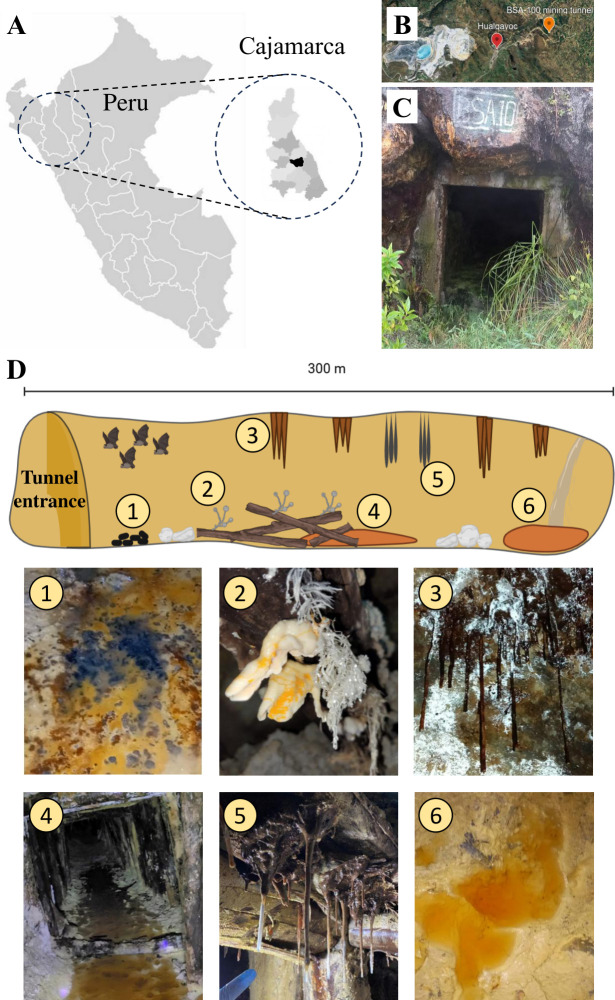
Location and characteristics of the BSA-100 mining tunnel site. Map of Peru highlighting the Cajamarca region and Hualgayoc district (black) (**A**). Location of the BSA-100 mining tunnel (6°45′27″S, 78°35′06″W) in the Hualgayoc district (**B**). (Panel B ©2026 Airbus, CNES/Airbus, Landsat/Copernicus, Maxar Technologies.) Entrance of the BSA-100 mining tunnel (**C**). Scheme of the internal section of the mining tunnel showing mineral and microbial arrangements (**D**): (1) sediments close to bat guano with black and orange spots; (2) fungal mycelia attached to wood sticks; (3) brown stalactites on the roof of the mining tunnel; (4) AMD stream with wood touching the sediment; (5) snottites hanging from wood on the roof of the mining tunnel; (6) a small orange-yellow pond produced by the AMD waterfall.

An abandoned mining tunnel (BSA-100) located in Hualgayoc, Cajamarca (Peru) was selected as the sampling site. Mining operations in this area started in 1940 by the Compañía Explotadora de Minas San Agustín (CEMSA) and ceased in 1971 (14). This tunnel is 300 m long with galleries and vent shafts that allow oxygen diffusion. AMD was found at two points in this tunnel: (i) as a shallow stream at 150 m (PM1) and (ii) as a waterfall at 300 m (PM2) (Fig. 1). Organic matter is present as bat guano and wood (building material), offering a natural source of complex organic matter for acidophilic microorganisms. The occurrence of black and orange spots on sediments and water interfaces, indicative of different biofilms or mineral composition, likely indicates active carbon, sulfur, and iron cycling. Other microbial signatures like fungal mycelia and snottites (hanging biofilms with pH < 1.0) are also visible throughout the tunnel, indicating the microbial diversity in this extreme habitat.

AMD and sediments were collected at PM1 and PM2 for chemical, mineralogical, and microbiological analyses. AMD was collected using a 500 mL glass bottle, and sediments were collected nearby by using 60 mL sterile cut syringes, which were introduced to a depth of 10 cm. The pH, oxidation-reduction potential (ORP), and temperature of water samples were measured *in situ* using a Hanna HI2002 multiparameter device (Hanna Instruments, USA) coupled to a pH electrode with a reference of Ag/AgCl (HI11310). The pH of sediments was measured using a 0.01 M CaCl_2_ solution at a 1:2 (sediment:CaCl_2_ solution) ratio. The total metal content (unfiltered) of water samples was analyzed by inductively coupled plasma optical-emission spectrometry (ICP-OES) at SGS Peru (https://www.sgs.com/es-pe) using a Thermo Scientific iCAP 7000 following the Method 200.8 (U.S. EPA, 1994). The mineral composition of sediment samples was analyzed in the Department of Geosciences at the University of Tübingen using powder X-ray diffraction (powder-XRD) analysis (see “Mineralogical analyses,” below, for details). Sediment cores were stored under anoxic conditions by using a 2.5 L jar and an AnaeroGen system (Thermo Scientific, USA) and used for microbiological analysis. These sediment samples were kept at 4°C and transported to the Laboratory of Biotechnology of the Universidad Nacional de Cajamarca (Peru).

### Enrichment of an aSRB consortium

An aSRB-containing consortium was enriched in microcosms (Fig. S1). Briefly, 100 mL glass serum bottles were filled with 50 mL of mineral salts medium (MSM) (15). The medium was supplemented with 0.1 g L^−1^ of yeast extract, 0.5 g L^−1^ of L-cysteine, and 1.42 g L^−1^ (10 mM) of Na_2_SO_4_ as an electron acceptor. Bicarbonate solution was eliminated from the basal composition, and the pH was adjusted to 2.5 by using 3 M HCl. Anoxic conditions in all microcosms were obtained by degassing the medium with N_2_ (99% purity) and by filling out the headspace with 1.5 atm of N_2_/CO_2_ (80:20 vol/vol) gas mixture into serum bottles previously sealed with butyl rubber septa and aluminum crimps. After sterilization by autoclave, glycerol was added to the medium as an electron donor and carbon source from a 1 M autoclaved anoxic stock to obtain a final concentration of 5 mM. Vitamin solution was sterilized by using a 0.22 μm pore-size polyethersulfone filter and then added to the medium. A sediment mixture was used as the initial inoculum. Sediment samples were added to the serum bottles with 50 mL of MSM and mixed at 10% (5 g/50 mL) concentrations. Inoculation (in triplicates) was performed inside a plastic bag under N_2_ atmosphere, with anoxic conditions verified using resazurin anaerobic strips (AnaeroTest strips, Oxoid, UK). Bottles were incubated statically in the dark at 30°C and monitored for pH and sulfide production using the methylene blue method every 4 days (16) (see “Analytical methods,” below, for details). Transfers to fresh medium were performed using enrichment cultures, maintaining the culture conditions as mentioned above. Three transfers were performed in total.

### AMD bioremediation experiment

For this experiment, a synthetic AMD (sAMD) medium was designed based on the average metal composition of the natural AMD found in the BSA-100 mining tunnel (Table 1). The experiment was performed in 1,000 mL Schott bottles containing 500 mL of sAMD. Briefly, MSM was supplemented with a 10-fold metal (MSS) and iron stock solutions (ISS). MSS (pH ~3.0) was prepared by adding salts to 500 mL of distilled water. ISS (pH ~3.0) containing 324.70 mM FeSO_4_.7H_2_O was prepared in anoxic water. Stocks were stored at room temperature. MSS was added first to MSM at 10% (vol/vol). Bottles were closed with rubber stoppers and red screw caps with central aperture (Duran, DWK Life Sciences, Germany). Anoxic conditions were obtained by degassing and then filling the headspace with 1.8 atm N_2_/CO_2_ (80:20 vol/vol) gas mixture. After autoclaving, a 10% (vol/vol) filter-sterilized ISS was added. The final sAMD mixture contained a theoretical value of 50.53 mM sulfate and a pH of 2.9. The bottles were then supplemented with vitamins, glycerol as a carbon source (25 mM), and with the previously enriched aSRB consortium (10%). Subsequently, more inoculum was added at days 32 and 86 to enhance sulfate reduction activity. A negative abiotic control (sAMD + glycerol) was included to distinguish biotic versus abiotic processes. Bottles were incubated in the dark at 30°C and monitored for 160 days for physicochemical parameters (pH, ORP, sulfide, sulfate, glycerol, and acetate) and up to day 200 for metal(loid) concentrations.

**TABLE 1:** Metal composition of natural AMD (BSA-100 mining tunnel) and sAMD

Cation	AMD		sAMD (mM)
	PM1 (mM)	PM2 (mM)	
Fe^2+^	21.11	43.83	40.25^*a*^
Al^3+^	5.29	5.49	5.39^*a*^
Zn^2+^	7.88	0.06	3.97^*a*^
Mn^2+^	6.26	0.01	0.07^*a*^
Mg^2+^	3.42	0.12	5.99^*b*^
As^3+^	0.07	0.21	0.14
K^+^	0.01	0.12	2.01^*b*^
Na^+^	0.03	0.02	5.56^*a*^
Cd^2+^	0.04	0.00	0.02^*b*^
Co^2+^	0.00	0.02	0.01^*b*^
Ni^2+^	0.01	0.02	0.01^*b*^

^*a*^ Using the sulfate salt for sAMD.

^*b*^ Using the chloride salt for sAMD.

### Analytical methods

For physicochemical analyses, samples were collected during the bioremediation experiment using sterile syringes. Bottles were shaken beforehand to obtain homogeneous samples. pH and ORP were measured in 2 mL of sample under an anoxic atmosphere using a multiparameter probe (Hanna Instruments, USA). Sulfide was determined using the methylene blue assay using 1 mL of sample mixed with 5% (wt/vol) ZnCl_2_ to stabilize the sulfide as Zn sulfide precipitates (16). Samples were analyzed on the same day of collection. Samples for dissolved sulfate and metal(loid)s (1 mL each) were filtered through a 0.22 μm pore-size polyethersulfone filter and then analyzed using a Dionex DX-120 Ion Chromatograph (Thermo, USA) and ICP-MS (Agilent 7900), respectively, with typical errors <10%. Organics (glycerol and acetate) were quantified by high-performance liquid chromatography (HPLC) in 1 mL of sample using Shimadzu Prominence HPLC, which was equipped with an LC-20AT solvent delivery unit, a CTO-10ASvp column oven, and a RID-20a refractive index detector. Measurements were performed weekly (pH, ORP, sulfide) and every 10 (sulfate and organics) and 20 days (metal(loid)s) throughout the experiment (160 days). An additional sampling point was added for metal(loid) measurement at day 200.

### Chemical modeling

The program PHREEQC interactive (https://www.usgs.gov/software/phreeqc-version-3) version 3.7.3.15968 was used for chemical calculations to predict the formation of mineral phases during the bioremediation experiment at days 0, 30, 60, 90, 120, and 160. Input data consisted of metal(loid)s and physicochemical parameters measurements at each day of the experiment using the minteq.v4 database.

### Mineralogical analyses

The mineralogy of initial sediments and experimental samples (day 120) was determined by XRD analysis. One gram of dried sediment samples was powdered in a mortar and pestle under oxic conditions for preparation for powder-XRD. Sediment samples were transferred onto a 51.5 mm Bruker PMMA ring (Bruker AXS GmbH, Karlsruhe) and analyzed on a Bruker D8 Advance powder diffractometer with the Cu Kα radiation at 40 kV/20 mA, 0.2 mm divergence slit, a fixed knife edge to suppress air scattering, and a VANTEC-1 detector in scanning mode. The diffractograms were acquired in a continuous scan range of 2–70 °2θ with the step size of 0.008°. For experimental samples, 2 mL of suspensions was centrifuged at 12,100 × *g* for 1 min to pellet the solid phases, which were then washed three times with Milli-Q water by the same centrifugation protocol. Minerals were dried for 1–3 days in the glovebox (Mbraun, 100% N_2_) and stored in anoxic conditions. The µXRD raw diffractogram was acquired with a Bruker D8 Discover GADDS XRD2 micro-diffractometer with a standard sealed tube and Co-anode (Co Kα radiation with 0.179 nm wavelength) at 30 kV/30 mA. Measurements were performed at two detector positions, 15° and 40° for 240 s. Diffractograms were analyzed with Match! Software (Crystal Impact, Bonn, v. 3.11.5.203) with the Crystallography Open Database (COD-Inorg REV211633 2018.19.25).

### Scanning electron microscopy and energy dispersive X-ray spectroscopy

Samples were collected for scanning electron microscopy (SEM) analysis at day 86 of the experiment using two preparation methods. Samples for cell analysis were prepared with a focus on preservation of cellular structure by fixing in 2.5% electron microscopy-grade glutaraldehyde overnight incubation at 4°. The next day, samples were washed three times with Milli-Q water, and a sequential ethanol dehydration and drying process was applied as detailed in reference 17. Samples for mineral analyses were dried onto carbon adhesive tabs attached to aluminum stubs in an anoxic chamber. Once dry, the samples were coated with 8 nm of platinum using a BAL-TEC SCD 005 sputter coater to reduce charging effects during analysis. Morphological characterization of experimental products was performed using a Crossbeam 550L SEM (Zeiss, Oberkochen, Germany) operating at an acceleration voltage of 2 kV and a working distance of 4.9 mm. All micrographs were taken using the Secondary Electron Secondary Ion (SESI) detector.

Energy dispersive X-ray spectroscopy (EDS) was performed to determine elemental composition of the samples by using a Zeiss Crossbeam 550L Scanning Electron Microscope equipped with an Oxford Instrument Energy Dispersive Spectrometer (UltimMax 100, Oxford Instrument, Abingdon, United Kingdom). EDS point and line scans were performed using an acceleration voltage of 15 kV and a probe current of 2.1–2.3 nA at 5 mm working distance, with a detector deadtime value of around 35%.

### Nucleic acid extraction and molecular analyses

#### Nucleic acid extraction

DNA was extracted from 2 mL samples (triplicates) at days 0, 30, 80, and 160 of the bioremediation experiment using the DNeasy PowerSoil kit (Qiagen, Frederick, Maryland, USA) and eluted using 50 µL of DNase/RNase-free water. After extraction, DNA was quantified using an Invitrogen Qubit 4 Fluorometer and 1× Qubit dsDNA High Sensitivity Assay Kit (ThermoFisher Scientific, Waltham, MA, USA). Total RNA was extracted from samples (triplicates) at days 0, 30, 60, 90, 120, and 160 of the bioremediation experiment according to (18). Briefly, DNA was removed using the TURBO DNA-Free kit (Invitrogen, USA), and successful DNA removal was confirmed by PCR using universal bacterial primers as described previously (18, 19). The RNA quality was evaluated by 1% (wt/vol) agarose gel electrophoresis and concentration quantified by spectrophotometry in a Qubit 2.0 Fluorophotometer (Invitrogen, USA). Complementary DNA (cDNA) was synthesized using 0.5 g of the previously isolated RNA. Reverse transcription reactions were performed with SuperScript II Reverse Transcriptase (Invitrogen, USA) following the manual instructions using random hexamer primers.

#### Quantitative PCR analysis

The SsO Advanced Universal SYBR Green Super Mix (Bio-Rad, USA) in CFX96 Touch Real-Time PCR Detection System (Bio-Rad, USA) was used to test a set of primers targeting the 16S rRNA gene (341 F [5′-CCTACGGGAGGCAGCAG-3′] and 797 R [5′-GGACTACCAGGGTATCTAATCCTGTT-3′]). Quantitative analysis of the samples was performed by triplicate using 10 µL as the total volume of PCR. PCR mixture was composed of 0.15 µL (5µM) of the forward primer, 0.45 µL (5µM) of the reverse primer, 5 µL of SYBR green (Invitrogen), 1 µL of cDNA as template, and nuclease-free water up to the final volume. The following amplification conditions: 95°C for 10 min, 40 cycles of 95°C for 30 s, 60°C for 30 s, and 72°C for 30 s were used for the amplification. After thermal cycling amplification, a melting curve was developed to check PCR specificity. A single predominant observed peak indicated the specificity of the PCR product (20). Standard curves were constructed with serial dilutions of known amounts of the 16S rRNA gene. Quantification of 16S rRNA copies was performed by comparing the amplification results to a standard dilution series ranging from 10^2^ to 10^9^.

#### 16S rRNA and metagenome sequencing

For 16S rRNA gene amplicon sequencing of samples of days 0, 30, and 80, library preparation steps and Illumina MiSeq sequencing (Illumina, San Diego, CA, USA) using the 2 × 250  bp MiSeq Reagent Kit v2 (500 cycles kit) were performed at the Institute for Medical Microbiology and Hygiene of the University of Tübingen.

16S rRNA Illumina-tag PCRs of samples from day 160 were performed on DNA extracts per the Earth Microbiome Project’s protocol (21). PCR products were pooled and gel-purified on a 2% agarose gel using the QIAquick Gel Purification Kit (Qiagen, Frederick, Maryland, USA). Before sequencing, the purified pool was quality-checked using an Agilent 2100 BioAnalyzer and Agilent DNA High-Sensitivity DNA kit (Agilent Technologies, Santa Clara, California, USA). The purified pool was stored at −20˚C and then sequenced by Wright Labs, LLC (Huntingdon, PA, USA) using an Illumina MiSeq v2 chemistry with paired-end 250 base pair reads.

DNA extract from a sample of day 160 was used to prepare metagenomic libraries using the Nextera XT DNA Library Preparation kit (Illumina, San Diego, CA, USA). Libraries were quality checked using an Agilent 2100 Bioanalyzer and DNA High-Sensitivity kit and then pooled in an equimolar ratio. The pool was gel-purified using a 2% agarose gel and the Qiagen QIAquick gel extraction kit (Qiagen, Germantown, MD, USA). Following purification, the pool was sequenced by Wright Labs, LLC (Huntingdon, PA, USA) using an Illumina NextSeq to produce 2 × 150 bp reads.

#### Bioinformatics

##### Processing of 16S rRNA sequences

Between 7,800 and 194,655 read pairs were obtained for each of the 9 samples (in total 757,935 read pairs) in two sequencing runs. Data processing, including quality control, reconstruction of sequences, and taxonomic annotation, was done using nf-core/ampliseq v2.12.0 (22, 23) of the nf-core collection of workflows (23). The pipeline was executed with Nextflow v24.04.4 (24) and Singularity v3.8.7 (25). Samples from each sequencing run were treated separately for preprocessing. Primers were trimmed with Cutadapt version 4.6 (26). Adapter and primer-free sequences were processed with DADA2 v1.30.0 (27) to eliminate PhiX contamination, trim reads (before median quality drops below 35; forward reads were trimmed at 232 bp and reverse reads at 209 bp), correct errors, merge read pairs, and remove PCR chimeras. Subsequently, the amplicon sequencing variants (ASVs) of two sequencing runs were merged, and 341 ASVs were obtained across all samples and sequencing runs. Barrnap v0.9 classified 225 ASVs as of bacterial or archaeal origin, the remaining 116 ASVs representing an average 0.1% read counts per sample (<0.41%), were deemed spurious and discarded. Taxonomic classification was performed with DADA2 and the Genome Taxonomy Database (GTDB) (28), release R09-RS220. Intermediate results were imported into QIIME2 version 2023.7.0 (29) for visualization and exploration. The final abundance table had 225 ASVs and 6,353 to 163,015 read counts per sample (total 631,100 read counts). Alpha rarefaction curves were produced with the QIIME2 diversity alpha-rarefaction plugin, which indicated that the richness of the samples had been fully observed.

##### Processing of metagenomic sequences

Processing of metagenomic reads (12,279,132 reads) was performed in Kbase (https://narrative.kbase.us/narrative/193500) (30). After quality-filtering FastQC v0.12 (31) and trimming with Trimmomatic v0.36 (32), 99.5% of the initial reads were taxonomically classified with KAIJU v1.9.0 (33). Metagenomic reads were de novo assembled with Megahit v1.2.9 (34), metaSPAdes v3.15.3 (35), and IDBA-UD v1.1.3 (36). Based on N50 and L50 results (Fig. S2), the metaSPAdes assembly was selected for downstream analyses. Two high-quality metagenome-assembled genomes (MAGs) were binned using CONCOCT v1.1 (37). Quality check of MAGs was conducted with CheckM v1.0.18 (38). The MAG’s functional annotation was conducted with RASTtk v1.073 (39). GTDB-Tk v2.3.2 (40) was used for the taxonomic classification of MAGs. A phylogenetic tree for the two bacterial MAGs was generated using Fast Tree2 (41).

## RESULTS

### Physicochemical characteristics of the AMD system

The mine water showed high acidity and oxidizing conditions (pH 1.56 and ORP +200 mV at PM1, pH 2.55 and ORP + 300 mV at PM2), similar to a previous survey at the same location (42). Iron (dissolved + suspended) was the most abundant metal in the system (Table 1). Other metal(loid)s were detected as well (from the most to the least abundant): Fe>Al > Zn>Mn > Mg>As > K>Na > Cd>Co > Ni, indicating high dissolution of accessory minerals such as chalcopyrite CuFeS_2_, pyrrhotite Fe_(1*−x*)_S, and arsenopyrite AsFeS_2_ (43).

Sediment samples were similar in terms of pH and ORP (pH 2.38 and ORP + 350 mV at PM1, pH 2.54 and ORP + 380 mV at PM2). The XRD analysis indicates that the sediments were dominated by quartz (SiO_2_) (PM1) or mixtures of quartz and jarosite (KFe_3_(SO_4_)_2_(OH)_6_) (PM2). Minor amounts of titanomagnetite (Fe_2_TiO_4_) may be present (Fig. S3). The presence of poorly crystalline minerals, such as clays, cannot be ruled out due to the poor sensitivity of XRD to these phases in complex mixtures.

### Enrichment of the aSRB consortium

The aSRB consortium was able to reduce sulfate with glycerol as the sole carbon source at low pH (Fig. S4 and S5). The generation of black metal sulfide minerals (i.e., FeS) in the microcosms indicated sulfate reduction coupled to glycerol oxidation. The lowest pH at which this metabolism was detected in the first enrichment was 2.27 ± 0.03, rising up to 5.47 ± 0.14 at the end of this process. In contrast, sulfide was below the detection limit during the enrichment. pH and sulfide increased with more transfers, reaching values up to 6.0 and 7.34 mM, respectively, after 16 days of incubation in the third transfer.

### AMD bioremediation

**Fig 2:**
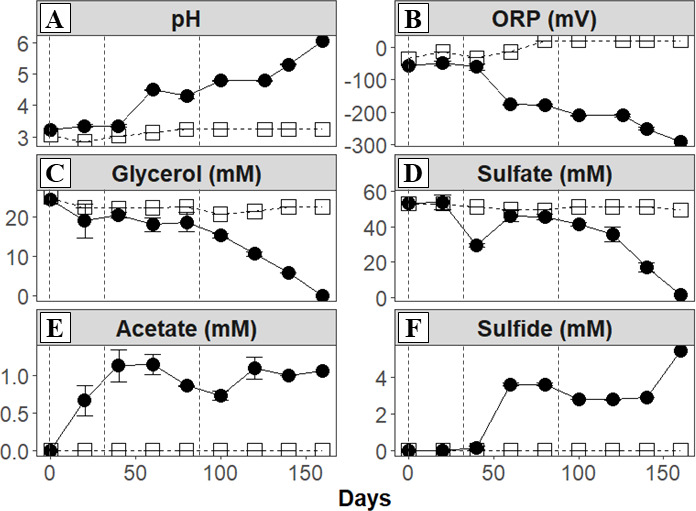
Physicochemical measurements throughout the AMD bioremediation experiment inoculated with the aSRB consortium. Measurements of pH (**A**), ORP (**B**), glycerol (**C**), sulfate (**D**), acetate (**E**), and sulfide (**F**) in biotic cultures (filled circle) and abiotic control (open square) over experimental time. Vertical dashed lines indicate time points at which inoculum was added (days 0, 32, and 86). Average and standard deviation of triplicates are displayed.

**Fig 3:**
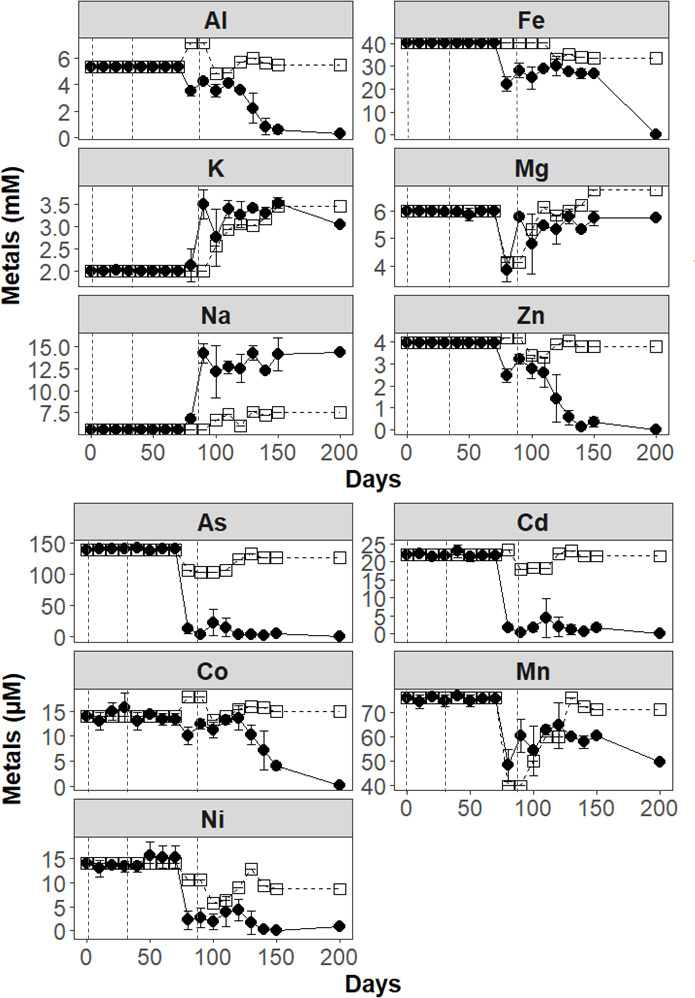
Concentration of dissolved metals (mM and µM) in biotic cultures (filled circle) and abiotic control (open square) of the AMD bioremediation experiment inoculated with the aSRB consortium. Note that Zn, Fe, Co, and Al were nearly completely precipitated at the end of stage 3 of the experiment (last sampling point, day 200) while other metals precipitated earlier (i.e., As, Cd, and Ni at day 80). Vertical dashed lines indicate time points at which inoculum was added (days 0, 32, and 86). Average and standard deviation of triplicates are displayed.

Physicochemical evolution over 160 days of the AMD bioremediation experiment (200 days for metal(loid)s) is displayed in Fig. 2 and 3. Three stages were visible during the bioremediation experiment and related to when inoculations were performed (days 0, 32, and 86). Stage 1 (from days 0 to 32) showed no marked changes in pH, ORP, and sulfide. Nevertheless, increases in glycerol (from 25 to 19.04 ± 3.51 mM) and acetate (from 0 to 0.67 ± 0.48 mM) suggested the onset of microbial activity (i.e., fermentation) at this time (Fig. 2). Metal(loid) concentrations remained unchanged during this part (Fig. 3).

Stage 2 (days 32–86) was triggered by re-inoculation of the aSRB consortium. pH, ORP, and sulfide reached values of 4.29 ± 0.05, −177.20 ± 1.70 mV, and 3.62 ± 0.05 mM, respectively, at the end of this stage. Sulfate, glycerol, and acetate decreased accordingly to 45.46 ± 1.27, 18.75 ± 1.91, and 0.58 ± 0.41 mM, respectively, throughout this time indicating active sulfate reduction by the aSRB consortium (Fig. 2). Dissolved Fe, Al, Zn, and Mn showed partial removal during this stage (25%–50% over respective total), while As, Cd, and Ni were completely removed from solution (Fig. 3).

Stage 3 of the bioremediation experiment (from days 86 to 160) was triggered by another re-inoculation, as sulfate reduction activity had ceased during the latter period of stage 2. At the end of stage 3, pH and ORP values reached values of 6.1 ± 0.02 and –290 ± 0.05 mV, respectively. Sulfide reached values of 5.43 ± 0.01 mM, indicating an increasing activity of the aSRB consortium. Sulfate and glycerol decreased completely by day 160 of the experiment. Low acetate concentrations were still observed at this point (ca. 1 mM) (Fig. 2). According to the last measurement (day 200), Zn and Cd decreased by 100%; Fe, Co, and As decreased by >99%; and Al and Ni decreased by ≥94%. Lower removal rates around 50% were obtained for Mn. Concentrations of K and Na showed notable increases (from 2 to 3.5 mM and 7–15 mM, respectively) after the second inoculum addition (for unclear reasons; see Text S1), while Mg remained at 6 mM with one outlier at day 70 (Fig. 3).

### Chemical modeling, minerals, and cell encrustation

Saturation index (SI) of minerals formed throughout the sAMD bioremediation is displayed in Table S1. Chemical modeling predicts the formation of various metal sulfides of Fe, As, Cd, Co, Ni, and Zn when dissolved sulfide is present. Minerals such as Al-sulfates were generally undersaturated, with the exception of alunite (KAl_3_(SO_4_)_2_(OH)_6_) and Al_4_(OH)_10_SO_4_ as the pH rose. The formation of Fe(III) minerals such as goethite (FeOOH) and jarosite (KFe_3_(OH)_6_(SO_4_)_2_) was possible when assuming a modest concentration of 0.1 mM for dissolved Fe(III). No Mn-containing minerals were expected to precipitate based on this modeling.

**Fig 4:**
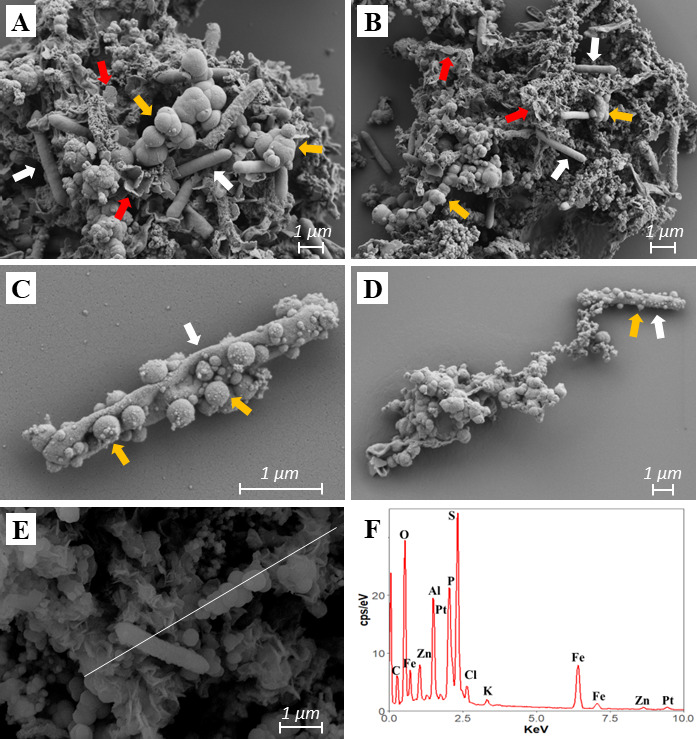
SEM images showing minerals and cell encrustation at day 86 of the AMD bioremediation experiment. Cell and mineral aggregates (**A and B**) and individual cells (**C and D**) with mineral encrustation at day 86. EDS line spectrum of solid phases demonstrating the occurrence of S-, Fe-, Al-, and Zn-containing minerals (**E and F**). The white line in panel E shows the line spectrum. Pt signals are from the coating. White arrows, microbial cells; red arrows, flake-shaped minerals; orange arrows, spherical minerals.

Cells and associated minerals on day 86 of the sAMD bioremediation experiment were investigated by SEM (Fig. 4). This time point, at the end of stage 2, coincided with the cessation of pH and sulfide increase and sulfate decrease. Furthermore, partial (Fe, Al, Zn, and Mn) and complete (As, Cd, and Ni) removal of metals were already observed. Spherical and flake-shaped minerals were the two main morphologies observed at this time (Fig. 4A and B). Cell encrustation was further observed (Fig. 4C and D). Spherical particles were occasionally attached to the cells, with EDS analysis indicating the presence of S and Al. Other elements such as Fe and Zn were also detected by EDS to be associated with the particles (Fig. 4E and F).

The XRD pattern of the final precipitates after 120 days is displayed in Fig. S3. The relatively noisy XRD pattern indicates contributions from low-crystallinity mineral phases. The signals at ~32° and 50° 2-theta cannot be confidently attributed to any phase typically precipitated during AMD bioremediation experiments such as poorly crystalline Al sulfates (e.g., hydrobasaluminite Al_4_(SO_4_) (OH)_10_·12–36H_2_O, felsöbányaite Al_4_(SO_4_) (OH)_10_·4H_2_O) and metal sulfides (FeS, Fe_3_S_4_, FeS_2_, ZnS), nor to any artifacts during drying (e.g., halite).

### 16S rRNA quantification and microbial community analyses

**Fig 5:**
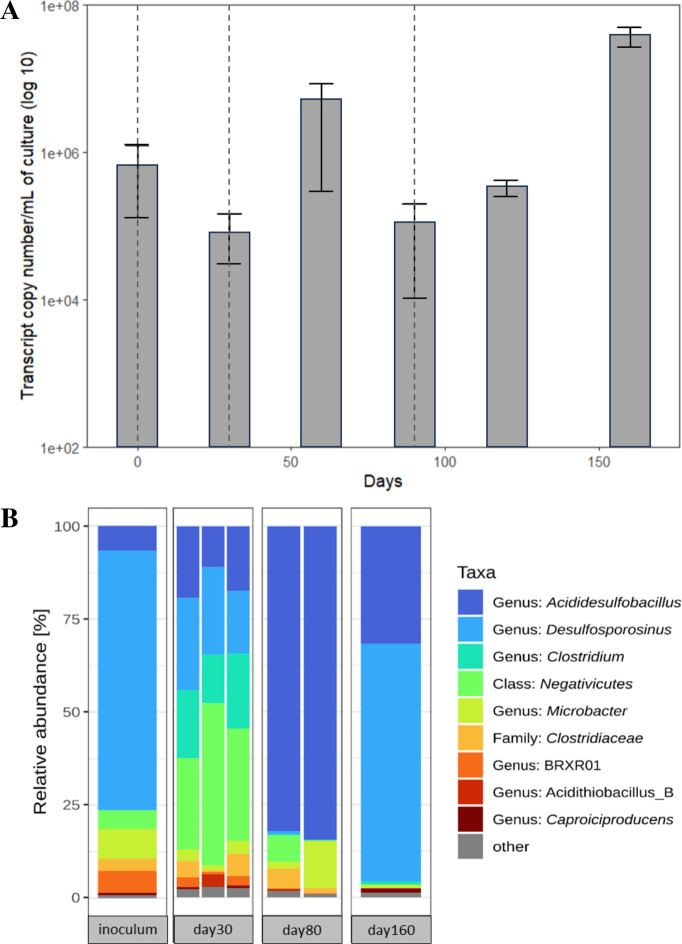
Quantification of microbial abundance (16S rRNA gene) (**A**) and microbial composition at the genus level of the inoculum at different time points (days 30, 80, and 160) (**B**) throughout the experiment. Vertical dashed lines indicate time points at which inoculum was added (days 0, 32, and 86). Average and standard deviation of triplicates are displayed. Taxa with less than 1% relative abundance in all samples were summarized as “other.”

Quantification of the 16S rRNA gene during the AMD bioremediation experiment is shown in Fig. 5A. 16S transcript copy numbers were around 6.7 × 10^5^ and 8.7 × 10^4^ copies/mL during days 0 and 30, respectively. An increase in bacterial copies up to 100-fold (up to 4.36 × 10^6^ copies/mL) was observed in the early phase of stage 2 from days 30 to 60, a timeframe featured not only by notable sulfate reduction activity but also by metal precipitation (i.e., As, Cd, and Ni). Despite the 10-fold decrease in gene copy numbers during further incubation from days 60 to 90 (from ~10^6^ copies/mL down to ~10^5^ copies/mL), an increase was noticed in the later stage from days 86 to 120 and to day 160, reaching a final value of 3.8 × 10^7^ copies/mL corresponding to the highest sulfate reduction activity and total metal precipitation.

Microbial community profiles at the genus level of the inoculum (aSRB consortium) and the AMD bioremediation experiment are shown in Fig. 5B. The inoculum was mainly composed of *Desulfosporosinus* (68.3%), *Microbacter* (8.0%), *Acididesulfobacillus* (6.5%), *Clostridiaceae* (5.9%), and *Negativicutes* (4.8%). Regarding the AMD bioremediation experiment, the microbial community at day 30 (*n = 3* replicates) showed the presence of *Negativicutes* (32.1% ± 10.05%)*, Desulfosporosinus* (21.4% ± 4.2%)*, Clostridiaceae* (15.8% ± 3.33%), and *Acididesulfobacillus* (7.2% ± 3.96%) as the predominant bacteria. Major changes were observed at day 80 of the experiment (*n = 2* replicates), with *Acididesulfobacillus* (79.9%–83.4%) as the most abundant genus followed by *Microbacter* (1.8%–12.5%), *Negativicutes* (7.1%–0.3%), *Clostridiaceae* (5.3%–1.5%), and *Desulfosporosinus* (0.5%–0.1%). At day 160 (*n = 1*), the microbial community changed further, with *Desulfosporosinus* (63.9%) and *Acididesulfobacillus* (31.6%) as key members at the end of the experiment (see Text S2).

### Metagenomics

Metagenomic analysis of the sample collected at day 160 showed a predominance of *Peptococcaceae-*related microorganisms (Fig. S6). Almost 60% of the quality-trimmed reads were taxonomically classified with KAIJU. From the classified reads, 96% were assigned to the *Firmicutes* phylum, 92% to the *Clostridia* class, 91% to the *Eubacteriales* order, and 85% to the *Peptococcaceae* family. At the genus level, the consortium was mainly composed of *Acididesulfobacillus* (58%), *Desulfosporosinus* (20%), and *Desulfitobacterium* (3%) (see Text S2). At the species level, *Acididesulfobacillus acetoxydans* had the highest relative abundance (58%), followed by *Desulfosporosinus acidiphilus* (8%), *Desulfosporosinus orientis* (6%), *Desulfosporosinus meridiei* (5%), and *Desulfitobacterium metallireducens* (0.9%). The remaining 2% of the classified reads were found to be part of the *Proteobacterium* phylum, mainly distributed as 0.7% *Gammaproteobacteria,* 0.4% *Deltaproteobacteria,* 0.2% *Alphaproteobacteria*, and 0.2% *Betaproteobacteria*. Within the *Deltaproteobacteria* organisms, *Desulfomonile tiedjei* was found at 0.03% relative abundance. Overall, the metagenomic analysis confirmed and complemented the taxonomic trends of the microbial community profiles obtained from 16S rRNA gene amplicon sequencing.

Two high-quality MAGs (003 and 013) were recovered from shotgun metagenomics at day 160. Taxonomic classification based on 115 bacterial marker genes showed affiliation of MAG003 to *Desulfosporosinus* sp. and MAG013 to *Acididesulfobacillus* sp. (Table S2). Both MAGs showed estimated completeness higher than 95% and contamination lower than 5%. The genome of MAG003 is bigger than that of MAG013, with 5,894 coding DNA sequences (CDS) and 4,676 CDS, respectively. MAG013 was related to *Acididesulfobacillus* sp001029295 with an average nucleotide identity (ANI) percentage of 96%. MAG003, however, was only classified up to the genus level with no specific organism at the species level closely related to it. A phylogenetic tree of the two bacterial MAGs confirmed the taxonomic classification based on GTDB-Tk (Fig. S7).

**Fig 6:**
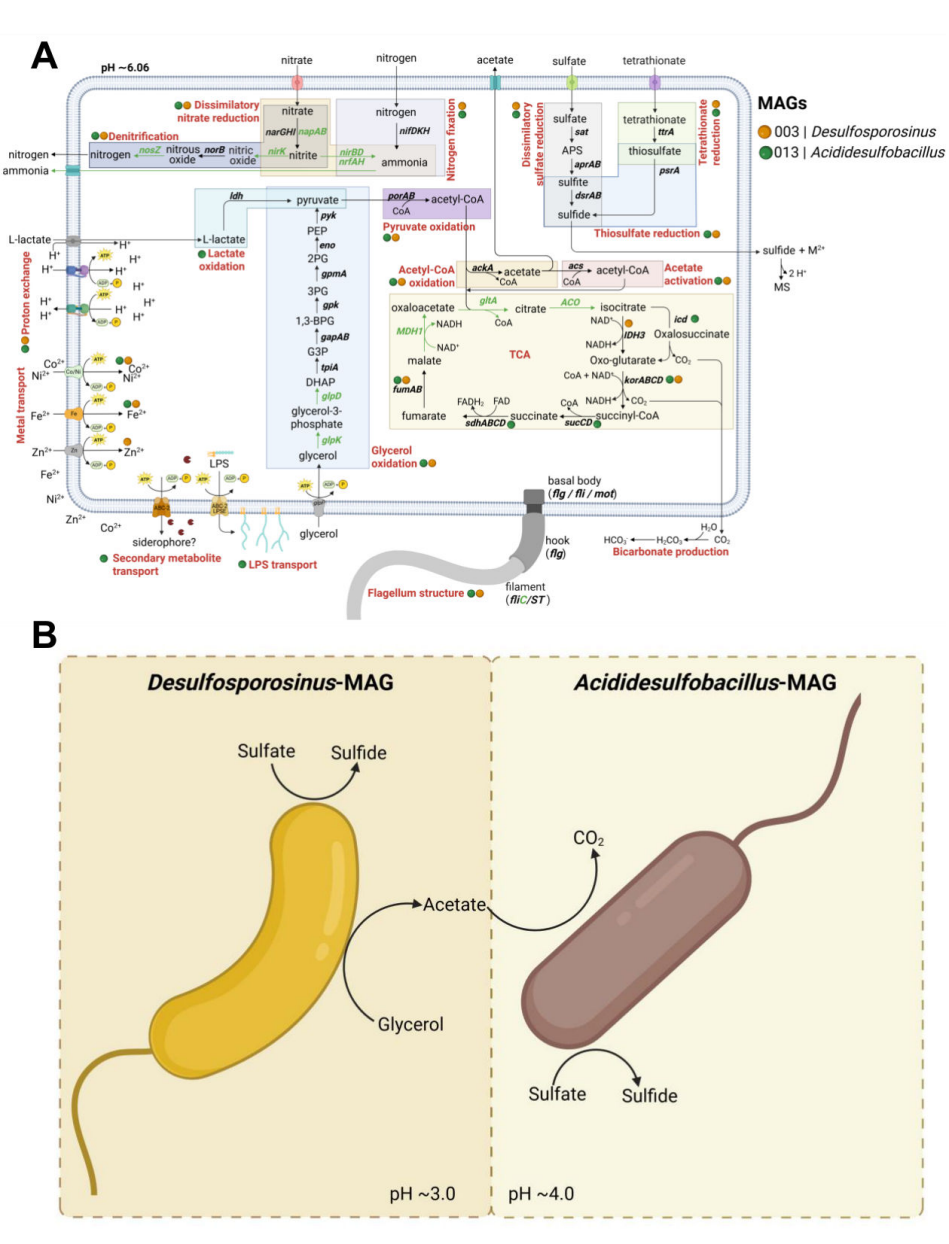
Conceptual model of metabolic pathways of the two MAGs identified in the aSRB consortium at the end of the AMD bioremediation experiment (**A**) and its potential syntrophic interaction (**B**). Blue and black circles indicate the presence of genes in MAG003 (Desulfosporosinus) and MAG013 (Acididesulfobacillus), respectively. Black names and arrows represent detected genes, while green names and arrows indicate absent genes. Motility-related structures (flagellar components) as well as transport systems for metal ions, protons, lipopolysaccharides (LPS), and secondary metabolites are depicted. APS, adenosine-5′-phosphosulfate; TCA, tricarboxylic acid cycle; CoA, coenzyme A; PEP, phosphoenolpyruvate; DHAP, dihydroxyacetone phosphate; ABC, ATP-binding cassette; MS, metal sulfide.

Both MAGs showed metabolic potential related to sulfur and carbon cycles (Fig. 6). Regarding the sulfur cycle, the occurrence of *sat, aprAB*, and *dsrAB* genes confirmed the presence of the whole dissimilatory sulfate reduction pathway in both MAGs. Moreover, the presence of the *ttrA* and *psrA* genes confirmed the capability for tetrathionate and thiosulfate reduction. Concerning carbon cycling, genes involved in glycerol oxidation (*tpiA*, *gapAB*, *gpk*, *gpmA*, *eno*, and *pyk*) were found in both MAGs. In addition, pyruvate-ferredoxin/flavodoxin oxidoreductase (*porAB*) and acetate kinase (*ackA*) coding genes of the acetyl-CoA pathway (pyruvate oxidation to acetate) were also found, confirming the incomplete carbon oxidation metabolic pathway in both of the obtained MAGs. Genes involved in acetate oxidation via the tricarboxylic acid (TCA) pathway (*icd*, *korABCD*, sucCD, sdhABCD, and fumAB) were found in *Acididesulfobacillus*-MAG, underscoring its complete oxidation pathway.

Genes related to an F type H^+^-ATPase and trehalose transport were found in MAGs (especially in *Acididesulfobacillus*-MAG), which would be of importance for proton exchange and osmotic balance, respectively, in acidophilic bacteria. Furthermore, Zn, Co/Ni, and Fe transporters were found in both MAGs, but the lack of detection of specific efflux pumps suggests no obvious metal detoxification mechanisms. Moreover, coding genes for ABC-type transports were found in *Acididesulfobacillus*-MAG with potential LPS and secondary metabolites (i.e., siderophore) transport roles.

## DISCUSSION

### The most pH-tolerant aSRB consortium enriched to date

An aSRB-containing consortium was enriched using AMD sediments (pH 2.38–2.54) with high metal(loid) concentrations (see Table 1) from the BSA-100 mining tunnel using glycerol as a carbon source. aSRB is an anaerobic prokaryotic group that performs the dissimilatory reduction of sulfate (or another oxidized sulfur compound) to sulfide as an end product under acidic conditions (6, 8, 44). Different electron donors/carbon sources have been coupled to this microbial metabolism, but sulfate reduction with glycerol (Reaction 1) is preferred due to its non-ionic nature and the fact that it cannot be deprotonated within the cytoplasm under acidic conditions, avoiding toxicity effects from cytoplasmic acidification (6, 8, 44, 45).


(1) $$\begin{array}{rl} & C_{3}H_{8}O_{3}\text{ (glycerol) }+0.75{SO}_{4}^{2-}+1.5H^{+}\to \\ & \;{CH}_{3}COOH\text{ (acetate) }+0.75H_{2}S+H_{2}{CO}_{3}+H_{2}O \end{array}$$

In our study, the aSRB consortium was active at an initial of pH 2.27 using glycerol, leading to an increase of pH (>5.0), sulfide production (~5.97 mM), and precipitation of black metal sulfides (Fig. S8) (8). Several aSRB consortia have been previously reported using acidic sediments as inoculum and glycerol at different pH ranges (at pH 3.0 (46), pH 3.3 (47), pH 3.4 (42), pH 3.8 (48), pH 4.0 (49), pH 4.2 (50), pH 4.5 (10, 51)). To our knowledge, the pH used in this study is the lowest pH value at which aSRB activity has been confirmed using glycerol as a carbon source. Our study, therefore, lowers the pH value threshold at which aSRB are thought to be active using glycerol as a carbon source.

### Biogeochemical evolution during different stages of the AMD bioremediation experiment

#### Stage 1—Initial fermentation

The bioremediation experiment using sAMD (pH 2.9) was performed to investigate the performance of aSRB consortium fed with glycerol as a carbon source for metal(loid) precipitation and pH neutralization. During the early incubation phase (days 0–32), no sulfate reduction activity was detected, coinciding with limited changes in pH, ORP, sulfide, and sulfate. This indicated a prolonged lag phase in sAMD medium compared to MSM medium. Similar extended lag phases have been previously reported in other surveys using aSRB and were mainly attributed to low pH and high metal concentrations (47), as well as to the amount and source of the inoculum (52, 53). Another factor to consider is the increased chloride ion concentration in our sAMD medium compared to MSM (see Table 1). Chloride has been shown to be toxic to some acidophiles (54) and may have contributed to the prolonged lag phase. Nonetheless, acidophiles are known to synthesize/transport trehalose to counteract osmotic imbalance to deal with chloride ions (55).

Interestingly, the concentrations of glycerol (from 24.33 to 19.04 mM) and acetate (from 0 to 0.67 mM) underwent changes (Fig. 2) in spite of the lack of sulfate reduction. This could be caused by fermentative metabolisms (i.e., *Clostridium*) as observed in other aSRB enrichments and AMD bioremediation experiments (10, 13, 42, 46, 56–58). Importantly, acetate generation via fermentation could contribute to the prolonged lag phase of aSRB, as this volatile fatty acid can be protonated at acidic pH, allowing it to pass across cell membranes where it can then acidify the cytoplasm (5, 6). In addition, yeast extract (0.1 g L^−1^ = ca. 0.05 g C/L = 4 mM C_org_) could also have contributed to the establishment of fermenters that were able to degrade complex carbon sources (e.g., *Clostridium*) (59). Hence, we hypothesize that fermentative metabolisms were active at this stage while sulfate reduction was not.

#### Stage 2—Rise of aSRB activity followed by cessation

During the second inoculation (stage 2: days 32–86), the activity of the aSRB consortium promoted a rise in pH and sulfide with a corresponding decrease in ORP and sulfate (Fig. 2). This resulted in partial (Fe, Al, Zn, and Mn) to complete (As, Cd, and Ni) metal removal (Fig. 3). Although black precipitates usually characterize sulfate reduction in AMD bioremediation experiments using aSRB, it is interesting to note that we observed primarily white precipitates at this stage (Fig. S8). These white precipitates were likely ZnS or Al hydroxysulfates (e.g., alunite or hydrobasaluminite) based on their characteristic coloration, occurrences in similar bioremediation experiments (47, 60), and their predicted formation based on PHREEQC modeling (Table S1). The increase in sulfide would also have clearly favored the precipitation of As, Cd, and Ni sulfides. Meanwhile, the FeS mineral was close to supersaturation, indicating slow and/or incomplete precipitation (Table S1). No Mn-bearing minerals were predicted to form, suggesting partial Mn removal either by adsorption or coprecipitation with other minerals.

We considered the different pathways that could contribute to sulfate removal (Text S3; Fig. S9). Sulfate reduction coupled to glycerol oxidation could only explain 22% of the sulfate removed at this stage. The precipitation of Al hydroxysulfates could only account for 11% of sulfate removal. To satisfy the sulfate mass balance, it was necessary to consider sulfate reduction coupled to the oxidation of acetate (30%; Reaction 2) and of yeast extract (maximum 32%). Thus, the consistently low acetate concentrations (<1 mM) in our experiments suggested rapid consumption of this organic compound. A minor percentage (5%) of the sulfate may be removed by sulfur comproportionation, a new metabolism recently discovered in acidophiles (Text S3).


(2) $${CH}_{3}COOH\text{ (acetate) }+{SO}_{4}{}^{2-}+2H^{+}\to H_{2}S+2H_{2}{CO}_{3}$$

Nonetheless, in spite of showing high sulfate reduction activity in the beginning of stage 2, this metabolism dropped at the end of this stage (pH decreased and no sulfide production; Fig. 2), possibly caused by waste products (H_2_S and acetate) or by cell encrustation (61–64).

#### Stage 3—Reinitiation of aSRB activity and comparison of metal removal efficiency to previous studies

During the third inoculation (stage 3: days 86–160), sulfate reduction started again (Fig. 2 and 3), highlighting the effectiveness of re-inoculation for restarting sulfate reduction activity. The formation of black precipitates coupled to the increase of sulfide was a clear indicator of metal sulfide formation (Fig. S8). Additional evidence of sulfate reduction during stage 3 was the pH increase and the further decrease of ORP (Fig. 2). We again considered the different pathways that could contribute to sulfate removal (Text S3; Fig. S9). At the end of stage 3, most of the sulfate removal could be explained by sulfate reduction coupled to the oxidation of glycerol (36%) or acetate (48%). Contributions from other metabolisms or precipitation as sulfate minerals were likely minor.

Metal(loid)s removal also characterized stage 3 of the AMD bioremediation experiment. At the end of this stage (last sampling point at day 200), the major elements Zn, Fe, and Al were removed up to 100%, 99.8%, and 94.4%, respectively, from solution (Fig. 3). This metal removal efficiency is consistent with the *K*_sp_ values of these elements which would promote their precipitation at pH values ≥4.5 (final pH at this stage was 6.06). High precipitation of Fe (204.99 mM Fe) was observed in an experiment treating AMD (pH 2.5) coupled with domestic wastewater (pH 7.4 to 8.9) as a carbon source using sediments from Rio Tinto as an aSRB-containing inoculum, in which >85% removal efficiency of this element was obtained after 118 days (65). As for Al, similar concentrations (1.5–4.5 mM) were removed in bioreactors inoculated with an aSRB consortium and glycerol as electron donor/carbon source that were fed with Al-laden synthetic acid mine water at a working pH value of 5.0 during 131 days of treatment (60). In addition, a similar removal efficiency of Zn (1.80 mM) was further observed in a seven-phase AMD treatment experiment (lower pH 2.8) using an up-flow anoxic packed-bed bioreactor inoculated with heavy metal-resistant immobilized SRB granules during 80 days of treatment, albeit with another electron donor (i.e., lactate) (66). Hence, our aSRB consortium removed metal(loids) at comparable or higher efficiency than previous treatments.

Minor elements such as Co and Mn were completely and partially removed, respectively, at the end of stage 3 (Fig. 3). Cobalt was likely removed as metal sulfides, as predicted by PHREEQC modeling (Table S1). Similar Co concentrations were removed from a synthetic acid mine water (pH 5) in a low pH sulfidogenic bioreactor inoculated with glycerol as a carbon source and immobilized aSRB during 462 days of treatment (13, 67). It is worth noting that although in our study, Mn was only partially removed (from 76.1 to 49.64 µM), several previous surveys reported a poor efficiency of removal of this metal in AMD bioremediation experiments using an aSRB as a higher pH is needed to form MnS (pH >7.0) (13, 67, 68). High Mn removal efficiency (up to 100%) has been observed for a mixture of mushroom compost and limestone during the treatment of artificial AMD (pH 2.2, Mn 10.32 mM) but similarly, possibly boosted by the high pH reached after treatment (pH >7.0) (69). In our experiment, Mn removal could have taken place in our cultures by the potential adsorption or coprecipitation with other minerals, as no Mn-bearing mineral is predicted to form via PHREEQC modeling.

### Re-inoculation as a strategy to overcome bioremediation limitations imposed by cell encrustation

Maintaining the continuous activity of aSRB during bioremediation is a challenge, given the cells’ exposure to high metals, low pH, and toxic waste products such as H_2_S and protonated organic compounds. In this study, we found cell encrustation as an additional limiting factor for aSRB activity. At the end of stage 2, in which sulfate reduction activity stopped, some cells were coated with nano-sized spherical minerals (Fig. 4). Similar structures rich in Al were found in enrichment cultures at pH 3.2–5.9 in which *Thermodesulfobium*-like bacteria dominated (47, 70). Another possibility is cell encrustation by FeS minerals, which is known to affect neutrophilic aSRB (62, 71). The small sizes of these minerals made it challenging to identify their specific compositions either by SEM-EDS or XRD.

Minerals attached to cell membranes are known to be detrimental to cell activity by limiting nutrient exchange (72), explaining the drop of sulfate reduction by our aSRB consortium at the end of stage 2 (73, 74). Cell encrustation is the product of biologically induced or influenced mineralization, in which metals (i.e., Fe^2+^ or Al^3+^) are attached to the outer part of microbial cells that serves as templates for metal binding and for the subsequent nucleation and growth of minerals (62, 71, 74–76). Since the presence of encrusted cells correlated with cessation of aSRB activity, we propose a direct link between the two. However, we stress that we could not confirm that the encrusted cells in our mixed cultures were aSRB. Cell encrustation has been mainly investigated in neutrophilic microorganisms. Therefore, reports of this process in acidophiles can open new research branches for improving AMD treatment processes.

It is noteworthy that re-inoculation successfully boosted the aSRB activity in our experiment, potentially coupled to shifts in the microbial community (e.g., shifts from being dominated by *Desulfosporosinus* to *Acididesulfobacillus*). Although we cannot rule out variations in the microbial community of the inoculum, we consider this unlikely, as the culture and incubation conditions for inoculum generation were consistent throughout the entire experiment. Multiple inoculation has been employed in similar AMD bioremediation experiments for improving low sulfate reduction rates (65). The purpose of re-inoculation is to increase the number of active microorganisms to counteract toxicity effects (77). Adding more active cells via re-inoculation allows for a sub-population to escape toxicity effects, to keep producing sulfide, and eventually to counteract this multi-toxicity by raising the pH and removing heavy metals. Some other strategies, such as protecting cells by using immobilized aSRB in porous beads manufactured from recycled glass or in granules conformed by polyvinyl alcohol, sodium alginate, and silicon sand, have been further used for increasing sulfate reduction activity during AMD bioremediation (13, 57, 58, 66, 78). Nonetheless, we found re-inoculation as an effective and simple strategy to restart aSRB activity.

### Synergistic interaction between aSRB populations for robust AMD bioremediation

The aSRB consortium used as inoculum in our study was mainly composed of *Desulfosporosinus* and *Acididesulfobacillus* (Fig. 5). *Desulfosporosinus* (68.3%) is able to oxidize glycerol to acetate (45). *Acididesulfobacillus* (6.5%), another aSRB, is able to completely oxidize acetate to CO_2_, which could be important for acetate removal after glycerol oxidation, thus avoiding cell inhibition under acidic conditions (79, 80). At stage 1 of the AMD bioremediation experiment, the microbial community was mainly composed of *Negativicutes* (32.1% ± 0.10%) and *Clostridiaceae* (15.8% ± 0.03%), two acidogenic bacteria usually reported in anaerobic culture experiments under acidic conditions. In our study, these bacteria could be responsible for glycerol fermentation with acetate as the main product (Fig. 2) (58, 81), correlating with the lack of sulfate reduction activity during this stage of the experiment. At stage 2, *Acididesulfobacillus* (81.7% ± 0.02%) was the predominant aSRB phylotype (Fig. 5), correlating with the constantly low concentrations of acetate and the increased sulfate reduction at this stage (Fig. 2). As far as we know, this is the first time in which *Acididesulfobacillus*-like taxa were observed to play a major role during AMD bioremediation. At stage 3, both *Desulfosporosinus* and *Acididesulfobacillus* dominated together (Fig. 5). The shift in the microbial composition during stages 2 and 3 of the AMD bioremediation experiment could have been promoted by re-inoculations but highlights the metabolic versatility of these bacteria to adapt to different carbon sources as well as to physicochemical variations.

MAGs affiliated with *Desulfosporosinus* sp. (MAG003) and *Acididesulfobacillus* sp. (MAG013) showed metabolic potential related to sulfur and carbon cycles (Fig. 6). The occurrence of genes related to sulfate (*sat, aprAB*, and *dsrAB*), thiosulfate (*psrA*), and tetrathionate (*ttrA*) reduction confirms the versatility of both MAGs to reduce different inorganic sulfur compounds (12, 82, 83). Nonetheless, tetrathionate metabolism has been mainly attributed to acidophilic sulfur-oxidizing bacteria (i.e., *Acidithiobacillus ferrooxidans*), in which the tetrathionate hydrolase enzyme catalyzes its conversion to thiosulfate, sulfur, and sulfate (84, 85). As for carbon cycling, the incomplete and complete carbon oxidation metabolisms of both MAGs suggested a synergistic interaction. While genes coding for glycerol, pyruvate, and acetyl-CoA oxidation (from glycerol to acetate) were found in both MAGs, genes for the TCA cycle (from acetate to CO_2_) were mainly found in *Acididesulfobacillus*-MAG (Fig. 6). Considering the capability to degrade different carbon sources of these MAGs, we propose that the *Desulfosporosinus*-MAG started sulfate reduction at pH ~3.0 using glycerol as a carbon source, producing acetate as a waste product. Once pH rose to ~4.0, the *Acididesulfobacillus*-MAG performed sulfate reduction by metabolizing acetate to CO_2_, thus avoiding acetate accumulation and therefore cell toxicity (Fig. 6). Previous research has shown a similar synergistic interaction, for example, between *Desulfosporosinus* and *Acidocella* (48). Our study shows, for the first time to our knowledge, a beneficial interaction between two aSRB.

### Conclusions

Uncontrolled discharges into the environment and the lack of reliable mitigation techniques have converted AMD into a worldwide environmental concern and, therefore, into one of the biggest challenges of the mining industry (1). The use of aSRB as a consortium instead of pure cultures for biological AMD treatment should be further explored, given the potential increase of cell growth and activity promoted by the metabolic redundancy and synergistic interactions in the consortium (9). Our work provided mechanistic insights into how the most acidic aSRB-containing consortium enriched to date was able to bioremediate a synthetic AMD solution. Using glycerol as the main carbon source, the pH rose to 6.1 with over 94% removal of various metals. Cell encrustation by minerals was identified as another potential toxicity mechanism that led to cessation of sulfate reduction. Multiple inoculations were employed as an effective and simple strategy to overcome these toxicity effects. Based on microbial community analyses, we hypothesized a synergistic interaction in which two aSRB—*Desulfosporosinus* and *Acididesulfobacillus*—create a coupled network of carbon flow, thus avoiding waste product accumulation and cell toxicity. Thus, our study showed the relevance of beneficial cooperation between aSRB to boost the biological treatment of AMD.

## Data Availability

Raw sequencing data have been deposited at NCBI in the Sequence Read Archive (SRA) under BioProject accession number PRJNA1217137. All data are presented within the article and its supplemental material. Additional requests for information can be submitted to the corresponding author(s).

## SUPPLEMENTAL MATERIAL

Supplemental material aem.00308-26-s0001.docx Fig. S1 to S8, Table S1, and Text S1 to S4.

Graphical abstract aem.00308-26-s0002.tiff Visual summary of the study.

ASM does not own the copyrights to Supplemental Material that may be linked to, or accessed through, an article. The authors have granted ASM a non-exclusive, world-wide license to publish the Supplemental Material files. Please contact the corresponding author directly for reuse.
