# Supplementary material for: Desulfosporosinus and Acididesulfobacillus dominate an acidophilic sulfate-reducing bacteria consortium during acid mine drainage bioremediation
Source: Appl Environ Microbiol. 2026 Apr 24;92(5):e00308-26. doi: 10.1128/aem.00308-26 (PMC13188906; doi:10.1128/aem.00308-26)
Supplement: Supplemental material — Fig. S1 to S8, Table S1, and Text S1 to S4. [file aem.00308-26-s0001.docx]

**SUPPLEMENTARY INFORMATION**


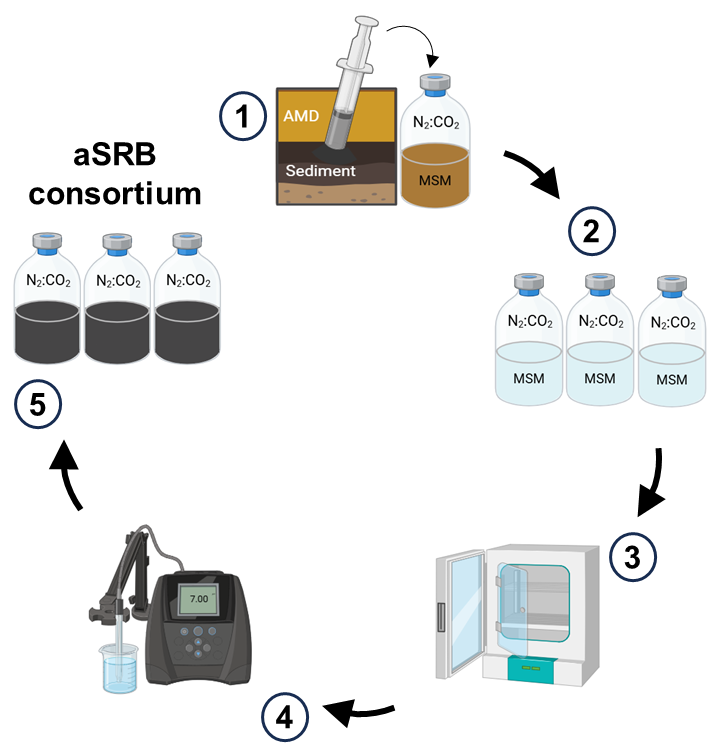


**Figure S1.** Enrichment and transfer setup to generate the aSRB consortium. 1. Inoculum for initial enrichment was prepared by mixing acidic sediments (PM1 and PM2) from the BSA-100 mining tunnel in mineral salts medium (MSM). 2. Enrichments cultures were set up in microcosms (triplicates) by transferring 10% of inoculum in MSM with glycerol as carbon source under acidic conditions. 3. Incubation of microcosms was at 30°C for 16 days. 4. Physicochemical parameters (pH and sulfide) of cultures were measured during incubation time. 5. Obtention of the aSRB consortium confirmed by the generation of blackish colors (metal sulfides precipitates). A transfer was initiated by adding the MSM with aSRB consortium (step 5) to fresh MSM (step 1) each time after 16 days of incubation (created with Biorender).


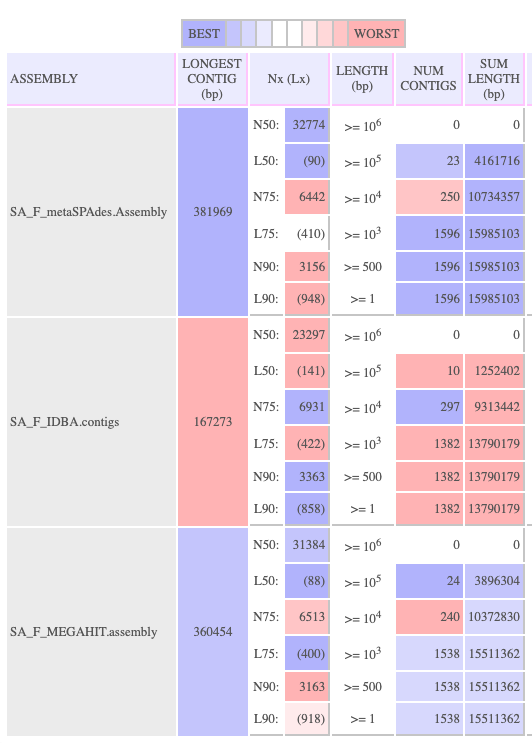


**Figure S2.** Distribution comparison of contigs assembled by three different tools (from top to bottom: metaSPAdes, IDBA, MEGAHIT). For downstream processing, the metaSPAdes assembly was chosen.


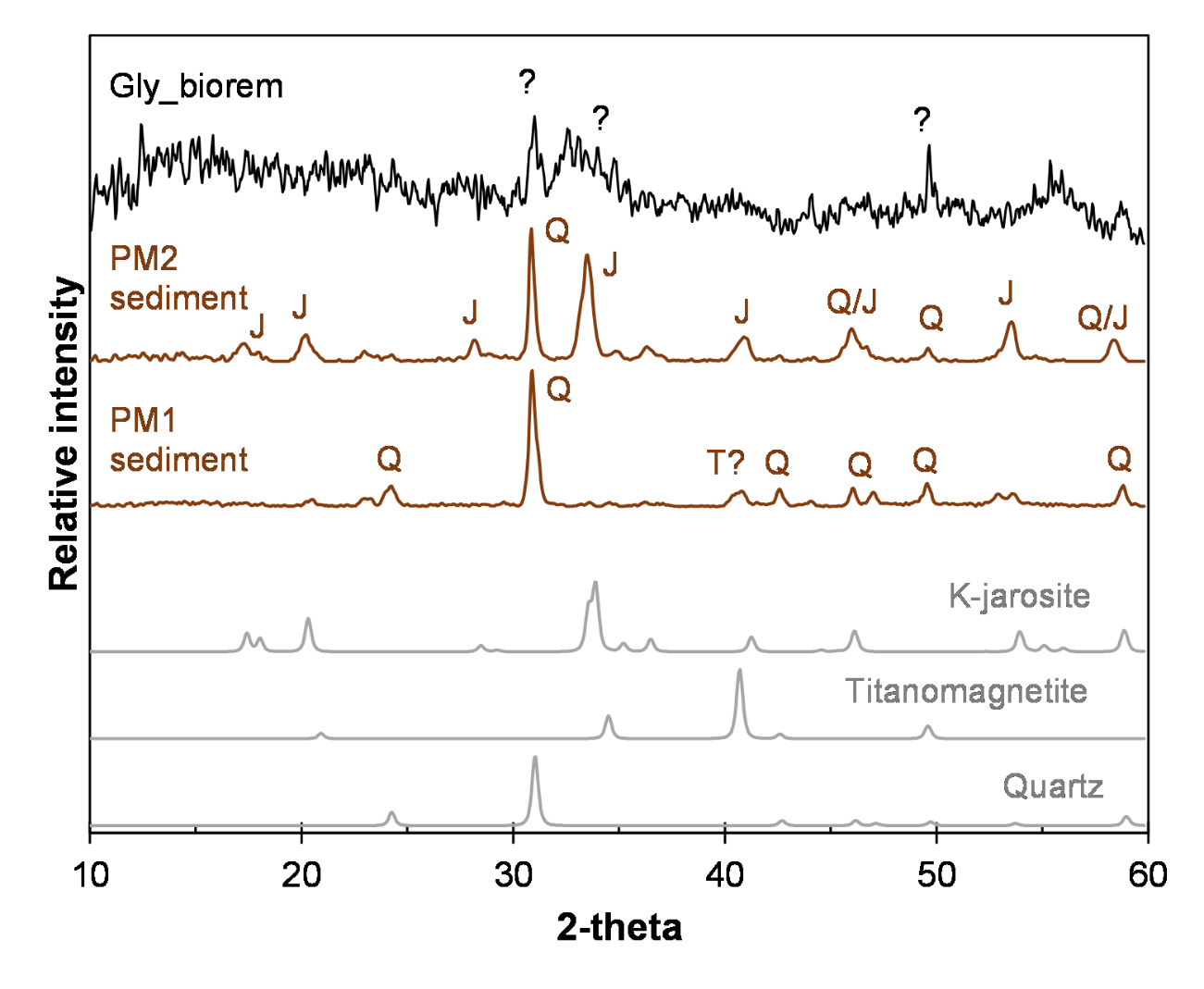


**Figure S3.** X-ray diffractograms of minerals in sediment samples and precipitates formed during the acid mine drainage (AMD) bioremediation experiment at day 120. Reference patterns in gray are quartz, Q (PDF# 96-900-0776), K-jarosite, J (96-155-7932) and titanomagnetite, T (96-900-0935). Gly_biorem: Bottle (replicate 1) with sAMD + aSRB consortium + glycerol; PM1-2: sediment from sampling point 1 and 2.


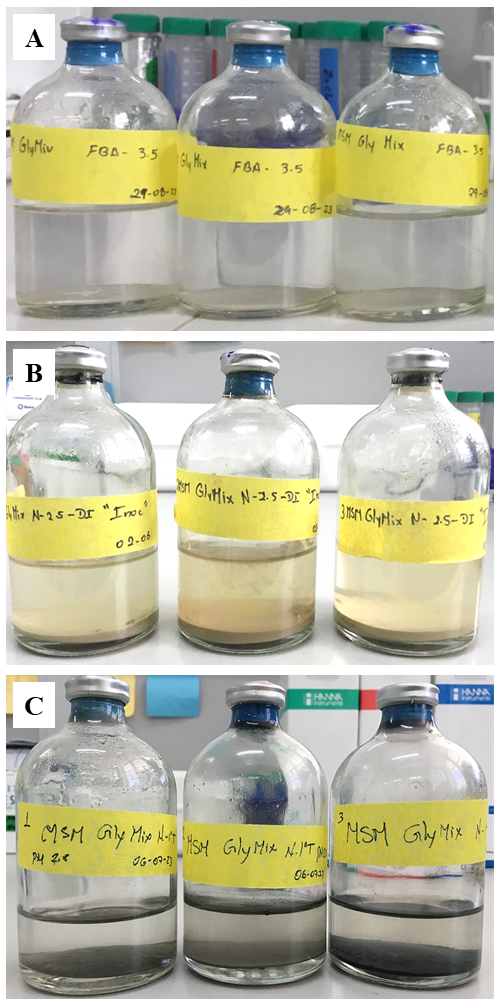


**Figure S4.** Visual changes of microcosms (triplicates) during the enrichment process of acidophilic sulfate-reducing bacteria (aSRB). Abiotic controls containing mineral salts medium (MSM) (A). Initial stage of microcosms showing yellowish inoculated sediments (B). Final stage of microcosms (after 16 days) showing black sulfide minerals (C).


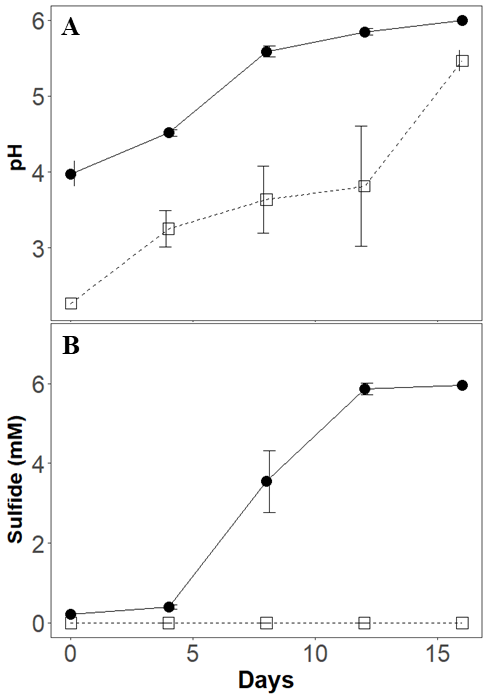


**Figure S5.** Physicochemical measurements of acidophilic sulfate-reducing bacteria (aSRB)-containing microcosms. pH (A) and sulfide (B) measurements of the initial enrichment (□) and within the third transfer (●) cultures using glycerol as carbon source after 16 days of incubation. Average and standard deviation of triplicates are displayed.

**
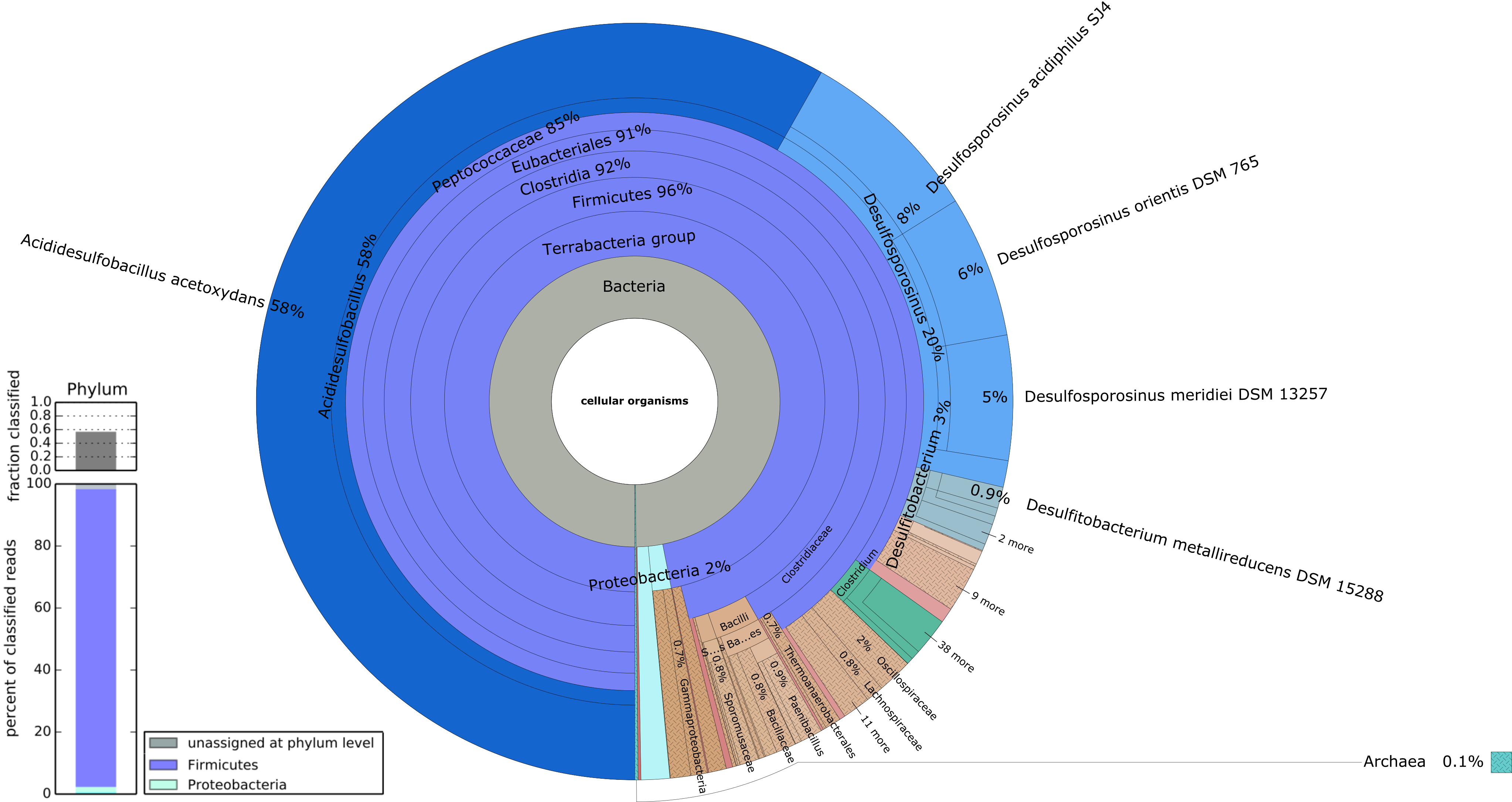
**

**Figure S6.** Taxonomic classification of metagenomic quality-filtered reads of sample taken at day 160 of the AMD bioremediation experiment. Figure modified from the Krona and bar plot original figures obtained when running KAIJU in Kbase (<https://narrative.kbase.us/narrative/193500>).


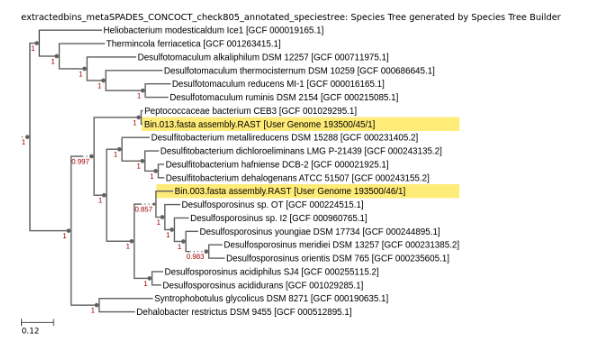


**Figure S7.** Phylogenetic tree for the two bacterial MAGs recovered from the aSRB consortium. Bin013 refers to the *Acididesulfobacillus-*MAG and Bin003 corresponds to *Desulfosporosinus-*MAG. The tree was generated by Species Tree Builder v2.2.0 in Kbase with default mode. *Peptococcaceae bacterium CEB3* from the NCBI taxonomy lineage corresponds to *Acididesulfobacillus* sp001029295 from the GTDB taxonomy lineage. The tree is rooted by *Heliobacterium modesticaldum* Ice1.


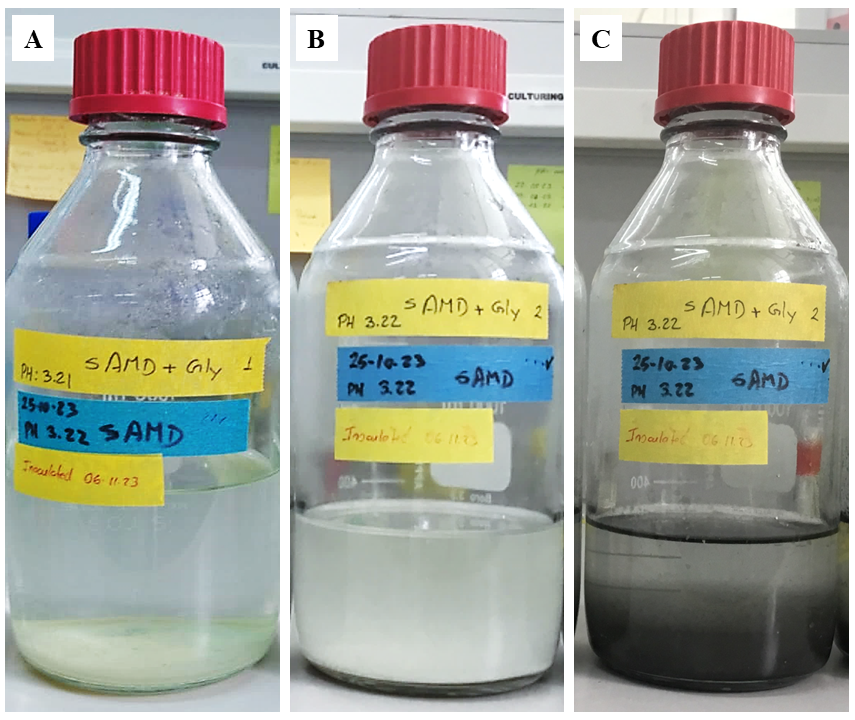


**Figure S8.** Visual changes of cultures during the incubation time of the AMD bioremediation experiment. Stage 1 (from 0 to day 32) showing green precipitates (A), stage 2 (from 32 to day 86) showing white precipitates (B), and stage 3 (from day 86 to 160) showing black precipitates (C). Average and standard deviation of triplicates are displayed.

**Table S1:** Saturation index (SI) of minerals formed throughout the synthetic acid mine drainage (sAMD) bioremediation experiment via Phreeqc modeling (minteq.v4 database). Input data consisted of metal(loid)s and physicochemical parameters measurements at day 0, 30, 60, 90, 120, 160. Fe (III) and Si were assumed to be at 0.1 mM concentrations. Blue is undersaturated while red is oversaturated, with the color gradient indicative of the degree of saturation.

| Element | Mineral | Days | | | | | |
| --- | --- | --- | --- | --- | --- | --- | --- |
|  |  | 0 | 30 | 60 | 90 | 120 | 160 |
| Al | Al(OH)_3_(am) | -5.22 | -5.55 | -1.36 | -2.19 | -0.81 |  |
|  | Al_2_O_3_ | -8.39 | -9.05 | -0.65 | -2.32 | 0.44 |  |
|  | Al_4_(OH)_10_SO_4_ | -10.14 | -11.24 | 2.73 | -0.15 | 4.29 |  |
|  | AlAsO_4_:2H_2_O | -7.47 | -8.02 | -2 | -3.54 | -3.3 |  |
|  | AlOHSO_4_ | 0.02 | -0.09 | 1.29 | 0.91 | 1.21 |  |
|  | Alunite | 1.23 | 0.57 | 8.91 | 7.25 | 9.84 |  |
|  | Bohemite | -2.98 | -3.31 | 0.89 | 0.05 | 1.43 |  |
|  | Diaspore | -1.32 | -1.65 | 2.55 | 1.71 | 3.1 |  |
|  | Gibbsite | -2.76 | -3.09 | 1.11 | 0.27 | 1.66 |  |
| Al/Fe | Hercynite | -7.33 | -8.21 | 2.98 | 0.73 | 4.58 |  |
| Al/K | K-Alum | -6.22 | -6.22 | -6.27 | -6.26 | -6.44 |  |
| As | Orpiment |  | 18.72 | 18.64 | 19.07 | 15.95 |  |
|  | Realgar |  | -2.8 | -4.52 | -4.27 | -6.53 |  |
| Cd | Greenockite |  | 3.63 | 4.88 | 3.43 | 2.91 |  |
| Co | CoS(alpha) |  | -3.23 | 0.09 | -0.02 | 1.38 |  |
|  | CoS(beta) |  | 0.4 | 3.72 | 3.61 | 5.01 |  |
|  | CoFe_2_O_4_ | 10.58 | 9.95 | 17.15 | 16.27 | 18.36 |  |
| Fe | FeS(ppt) |  | -4.31 | -0.91 | -1.16 | 0.32 |  |
|  | Mackinawite |  | -3.69 | -0.29 | -0.54 | 0.94 |  |
|  | Pyrite |  | 14.1 | 20.86 | 20.52 | 23.42 |  |
|  | Goethite | 2.63 | 2.39 | 4.63 | 4.42 | 4.96 | 6.3 |
|  | Greigite |  | 9.54 | 19.21 | 19.52 | 23.28 |  |
|  | H-Jarosite | 4.66 | 4.4 | 5.48 | 5.75 | 5.22 | 2.48 |
|  | Hematite | 7.67 | 7.21 | 11.69 | 11.25 | 12.35 | 15.01 |
|  | K-Jarosite | 7.45 | 7.09 | 9.55 | 9.75 | 9.83 | 6.92 |
|  | Lepidocrocite | 1.57 | 1.34 | 3.58 | 3.36 | 3.91 | 5.24 |
|  | Magnetite | 7.22 | 6.54 | 13.82 | 12.8 | 14.98 |  |
|  | Maghemite | -0.5 | -0.96 | 3.51 | 3.08 | 4.17 | 6.84 |
|  | Na-Jarosite | 4.36 | 4 | 6.46 | 6.86 | 6.92 | 4.2 |
|  | Ferrihydrite | -0.04 | -0.27 | 1.97 | 1.75 | 2.3 | 3.63 |
| Mg | Brucite |  | -13.18 | -10.4 | -10.91 | -9.78 |  |
|  | Epsomite |  | -2.83 | -2.86 | -2.91 | -2.87 |  |
|  | Magnesioferrite |  |  |  |  | 1.24 | 5.73 |
| Mn | MnS(grn) |  | -10.06 | -6.66 | -6.84 | -5.36 |  |
|  | MnS(pnk) |  | -13.32 | -9.92 | -10.1 | -8.62 |  |
| Na | Thenardite | -7.09 | -7.09 | -7.14 | -6.41 | -6.33 | -9.66 |
| Ni | NiS(alpha) |  | -4.95 | -1.52 | -2.52 | -1.12 |  |
|  | NiS(beta) |  | 0.55 | 3.98 | 2.98 | 4.38 |  |
|  | NiS(gamma) |  | 2.25 | 5.68 | 4.68 | 6.08 |  |
| S | Sulfur |  | 5.22 | 8.58 | 8.49 | 9.91 | 13.03 |
| Si | Quartz | -0.05 | -0.05 | -0.05 | -0.05 | -0.05 | -0.06 |
|  | Halloysite |  |  | 1.23 | -0.45 | 2.32 |  |
|  | Kaolinite |  |  |  |  | 4.36 |  |
| Zn | Sphalerite |  | 3.19 | 6.36 | 6 | 5.11 |  |
|  | Wurtzite |  | 0.72 | 3.88 | 3.53 | 2.64 |  |
|  | ZnS(am) |  | 0.84 | 4 | 3.65 | 2.76 |  |

**Table S2.** Principal characteristics of the metagenome-assembled-genomes obtained from the metagenome of the sample taken at day 160 of the AMD bioremediation experiment.

| MAG | GTDB-Taxonomy | Completeness | Contamination | ANI | Genome Size (bp) | N50 | GC% | CDS | tRNA | CRISPR arrays |
| --- | --- | --- | --- | --- | --- | --- | --- | --- | --- | --- |
| 003 | d__Bacteria;  p__Bacillota_B;  c__Desulfitobacteriia;  o__Desulfitobacteriales;  f__Desulfitobacteriaceae;  g__Desulfosporosinus;  s__ | 99% | 2% | - | 5609129 | 55448 | 42 | 5894 | 53 | 5 |
| 013 | d__Bacteria;  p__Bacillota_B;  c__Desulfitobacteriia;  o__Desulfitobacteriales;  f__Desulfitobacteriaceae;  g__Acididesulfobacillus;  s__Acididesulfobacillus sp001029295 | 98% | 4% | 96% | 4459274 | 171043 | 53 | 4676 | 55 | 3 |

**Text S1: Increase in dissolved Na and K at the beginning of stage 3**

We noted an increase in Na and K after the addition of the 2^nd^ inoculum. We therefore considered whether Na and K could be sourced from the inoculum or from any minerals undergoing dissolution (e.g., Na- & K-bearing clays) but considered these to be unlikely based on the known medium composition and the absence of any clay signal in the XRD. We also considered analytical errors in the ICP-MS but found no reason to exclude the data. Hence, we could not offer any explanation for this observation. Nonetheless, it is clear that other heavy metals (e.g., Fe, Al, Zn, Co, Ni, As) important for bioremediation consideration were removed successfully in this experiment.

**Text S2: Differences between the relative abundance of bacteria by 16S rRNA and metagenomics approaches.**

Differences in bacterial abundance obtained from 16S rRNA and metagenomic sequencing were noticed at day 160 in our study. Both approaches report relative abundances of microorganisms rather than true cell counts. In the case of the 16S rRNA dataset analyzed with nf-core/ampliseq, relative abundances may be influenced by factors such as rRNA gene copy number, primer binding efficiency, PCR amplification bias, and taxonomic classification. In contrast, relative abundances derived from metagenomic data analyzed with KAIJU can be affected by genome size, gene density, database representation, and read mappability. Despite these methodological biases, both approaches consistently indicate the dominance of *Acididesulfobacillus* and *Desulfosporosinus* in the sample, which supports the main conclusion of our study.

**Text S3: Sulfate mass balance calculations**

In the bioremediation experiments, sulfate could be removed via five different pathways:

1. Sulfate reduction with glycerol (by *Desulfosporosinus and Acididesulfobacillus*)

- 25 mM glycerol was supplied as the original carbon source

C_3_H_8_O_3_ (glycerol) + 0.75 SO_4_^2-^ + 1.5 H^+^ →

CH_3_COOH (acetate) + 0.75 H_2_S + H_2_CO_3_ + H_2_O **(1)**

1. Sulfate reduction with acetate (by *Acididesulfobacillus*)

- Acetate was produced as a by-product of Reaction 1 and can further fuel sulfate reduction

CH_3_COOH (acetate) + SO_4_^2-^ + 2 H^+^ → H_2_S + 2 H_2_CO_3_ **(2)**

1. Sulfate reduction with yeast extract (simplified to “CH_2_O”)

- The medium contained 0.1 g/L yeast extract (4 mM carbon), which could potentially be used by aSRB

CH_2_O + SO_4_^2-^ + 2 H^+^ → H_2_S + H_2_CO_3_ **(3)**

- For our calculations, we assumed all yeast extract to be consumed for aSRB to be conservative

1. Precipitation as Al hydroxysulfates

- The decrease of Al and the formation of white precipitates suggest the precipitation of these amorphous minerals. Alunite [KAl_3_(SO_4_)_2_(OH)_6_] and felsöbányaite [Al_4_(SO_4_)(OH)_10_·4H_2_O] are potential products.

3 Al^3+^ + 2 SO_4_^2-^ + K^+^ + 12 H_2_O → KAl_3_(SO_4_)_2_(OH)_6_ + 6 H^+^ **(4a)**

4 Al^3+^ + SO_4_^2-^ + 14 H_2_O → Al_4_(SO_4_)(OH)_10_·4H_2_O + 10 H^+^ **(4b)**

- For our calculations, we assumed a 3:2 ratio of Al:SO_4_ to be conservative.

1. Sulfur comproportionation

- Recent discoveries have suggested that acidophilic bacteria can couple the anaerobic oxidation of sulfide with sulfate as the electron acceptor (Amend et al., 2020; Aronson et al., 2023). The responsible bacteria – *Acidithiobacillus thiooxidans* – were identified in our consortium at low abundances of 0.33-1.18%.

3 H_2_S + SO_4_^2-^ + 2 H^+^ → 4 S^0^ + 4 H_2_O **(5)**

- We consider this metabolism to be the least likely to satisfy the sulfate mass balance in our experiment but included it for the sake of completeness. We attributed any remaining unexplained sulfate removal to this metabolism.

**References:**

Amend, J. P., Aronson, H. S., Macalady, J., & LaRowe, D. E. (2020). Another chemolithotrophic metabolism missing in nature: sulfur comproportionation. Environmental Microbiology, 22(6), 1971–1976. https://doi.org/10.1111/1462-2920.14982

Aronson, H. S., LaRowe, D. E., Macalady, J. L., & Amend, J. P. (2023). Isolation of a putative sulfur comproportionating microorganism. BioRxiv, 2023.06.08.544259. https://doi.org/10.1101/2023.06.08.544259

Knowing the amount of glycerol and Al removed, we can estimate the amount of sulfate removed by the various pathways at the end of stage 2 and stage 3 (**Figure S8**).


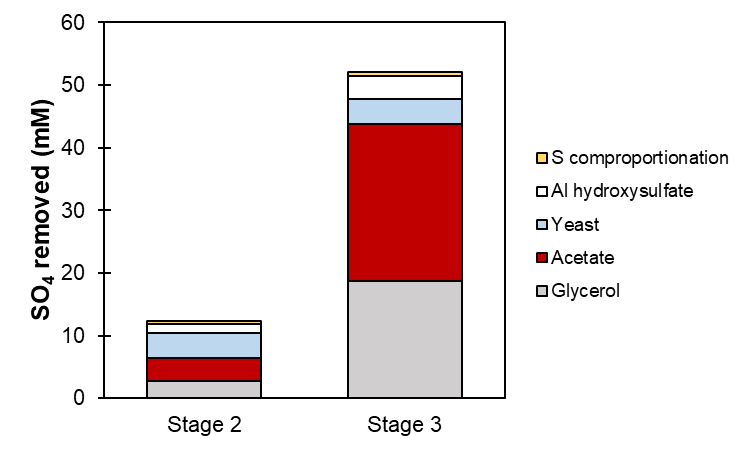


**Figure S9:** Estimates of the contributions of the different sulfate removal pathways at the end of stage 2 and 3 of the bioremediation experiment.

**Text S4: Metal adsorption by bacterial biomass calculations**

Considering a living cell number of 3.8 x 10^7^ cells/mL (according to cell quantification (see result section)) and the mass of a Gram-negative living bacterial cell (like *Desulfosporosinus*) of 1.048 x 10^-12^ g (Lewis et al., 2014), the total biomass will be:

- *3.8 x 10^7^ cells/mL x 1.048 x 10^-12^ g = 3.99 x 10^-5^ g biomass/mL*

As this result is in biomass per mL, we need to calculate the total biomass in the entire system (500 mL):

- *3.99 x 10^-5^ g biomass/mL x 500 mL = 19.95 x 10^-3^ g biomass*

According to Santos Alves et al. (2023), dry biomass of bacteria affiliated to *Pseudomonas* (Gram-negative) is able to adsorb 200 mg Zn/g of biomass, so:

- *1 g dry biomass ----- > 200 mg Zn*

*19.95 x 10^-3^ g biomass ---- > X*

- *X = (19.95 x 10^-3^ g biomass x 200 mg Zn) / 1 g biomass*
- *X = 3.99 mg Zn*

Therefore, 3.99 mg is the concentration of Zn potentially adsorbed in our system. In mM, it will be:

- *3.99 mg Zn/65.38g/mol = 0.061 mM Zn removed by adsorption.*

As we inoculated twice, we could multiply this value per 2.

- *0.061 mM Zn x 2 = 0.122 mM Zn*

Therefore, 0.12 mM Zn is the hypothetical concentration of Zn removed by the cell biomass of our AMD bioremediation system.

Following the same calculations, but this time for a Gram-positive living bacterial cell (cell mass = 0.648 x 10^-12^ g), the hypothetical concentration of Zn removed by the cell biomass (considering the same cell number of 3.8 x 10^7^ cells/mL) will be 0.08 mM Zn.

**References:**

Lewis CL, Craig CC, Senecal AG. Mass and density measurements of live and dead Gram-negative and Gram-positive bacterial populations. Appl Environ Microbiol. 2014 Jun;80(12):3622-31. doi: 10.1128/AEM.00117-14. PMID: 24705320; PMCID: PMC4054131.

Alves, D. A. S., Botelho Junior, A. B., Espinosa, D. C. R., Tenório, J. A. S., & Baltazar, M. dos P. G. (2023). Copper and zinc adsorption from bacterial biomass - possibility of low-cost industrial wastewater treatment. Environmental Technology, 44(16), 2441–2450. https://doi.org/10.1080/09593330.2022.2031312
